# Assessment of the Relationship Between Ambient Temperature and Home Blood Pressure in Patients From a Web-Based Synchronous Telehealth Care Program: Retrospective Study

**DOI:** 10.2196/12369

**Published:** 2019-03-04

**Authors:** Ching-Chang Huang, Ying-Hsien Chen, Chi-Sheng Hung, Jen-Kuang Lee, Tse-Pin Hsu, Hui-Wen Wu, Pao-Yu Chuang, Ming-Fong Chen, Yi-Lwun Ho

**Affiliations:** 1 Telehealth Center National Taiwan University Hospital Taipei Taiwan; 2 Department of Internal Medicine National Taiwan University Hospital Taipei Taiwan; 3 Department of Nursing National Taiwan University Hospital Taipei Taiwan; 4 Graduate Institute of Clinical Medicine, Division of Cardiology Department of Internal Medicine National Taiwan University Hospital and National Taiwan University College of Medicine Taipei Taiwan

**Keywords:** ambient temperature, home blood pressure, antihypertensive agents, retrospective studies

## Abstract

**Background:**

Decreased ambient temperature significantly increases office blood pressure, but few studies have evaluated the effect of ambient temperature on home blood pressure.

**Objective:**

We aimed to investigate the relationship between short-term ambient temperature exposure and home blood pressure.

**Methods:**

We recruited patients with chronic cardiovascular diseases from a telehealth care program at a university-affiliated hospital. Blood pressure was measured at home by patients or their caregivers. We obtained hourly meteorological data for Taipei (temperature, relative humidity, and wind speed) for the same time period from the Central Weather Bureau, Taiwan.

**Results:**

From 2009 to 2013, we enrolled a total of 253 patients. Mean patient age was 70.28 (SD 13.79) years, and 66.0% (167/253) of patients were male. We collected a total of 110,715 home blood pressure measurements. Ambient temperature had a negative linear effect on all 3 home blood pressure parameters after adjusting for demographic and clinical factors and antihypertensive agents. A 1°C decrease was associated with a 0.5492-mm Hg increase in mean blood pressure, a 0.6841-mm Hg increase in systolic blood pressure, and a 0.2709-mm Hg increase in diastolic blood pressure. This temperature effect on home blood pressure was less prominent in patients with diabetes or hypertension. Antihypertensive agents modified this negative effect of temperature on home blood pressure to some extent, and angiotensin receptor blockers had the most favorable results.

**Conclusions:**

Short-term exposure to low ambient temperature significantly increased home blood pressure in patients with chronic cardiovascular diseases. Antihypertensive agents may modify this effect.

## Introduction

### Background

Exposure to extreme temperatures, either high or low, is known to significantly increase mortality, and most temperature-attributable death is due to low, rather than high, ambient temperatures [1,2]. A previous study also documented the short-term effects of ambient temperature on morbidity from noncommunicable diseases [3]. Since it was first reported by Rose in 1961 [4], seasonal variation in blood pressure has been observed by many investigators, and this phenomenon is evident in a variety of patient populations [5,6]. Later studies confirmed that ambient temperature contributes to this phenomenon, and that decreased ambient temperature significantly increased office blood pressure [7-10]. Hypertension is an important risk factor for many cardiovascular diseases [11-13], and thus ambient temperature–associated fluctuations in blood pressure may be the underlying mechanism driving increased mortality and morbidity.

Recently, a Japanese study showed that home blood pressure (HBP) may be an even more important parameter than office blood pressure for predicting future coronary artery disease (CAD) and stroke events [14]. Few studies have investigated seasonal variations in HBP, and the existing studies have been on a small scale and examined elderly normotensive participants as the patient populations [15,16]. A recent large-scale Japanese population study using a Web-based platform also confirmed seasonal variations in HBP [17].

### Objective

We aimed to investigate the relationship between short-term ambient temperature exposure and HBP in patients with chronic cardiovascular diseases using a Web-based platform.

## Methods

### Study Design

This was a single-center, retrospective study and was approved by the Institutional Review Board of National Taiwan University Hospital, Taipei, Taiwan. We obtained informed consent from all participants.

### Patient Population

We conducted this study from January 2009 to December 2013 at the Telehealth Center of National Taiwan University Hospital. Patients older than 20 years with chronic cardiovascular diseases who were receiving telehealth care at our telehealth center were enrolled in the study group. Chronic cardiovascular diseases included (1) CAD with or without percutaneous coronary intervention, (2) prior myocardial infarction (MI), (3) heart failure, (4) peripheral artery disease, (5) prior stroke, and (6) hypertension. Patients who did not reside in Taipei during the study period were excluded. The decision on whether to receive the telehealth program was made by patients, their caregivers, or both.

### Telehealth Care Program

According to the classification of 4 generations of telemedicine in heart failure proposed by Anker et al [18], the telehealth care program used in this study was a fourth-generation telehealth program: it was a synchronized and integrated remote management program for chronic medical conditions. The internet-based platform was developed by the Graduate Institute of Biomedical Electronics and Bioinformatics, National Taiwan University, Taiwan. The program details have been reported previously [19-22]. Briefly, the telehealth program consisted of 4 key components: (1) biometric data, including single-lead electrocardiography, blood pressure, heart rate, and oxygen saturation, were transmitted from the patients’ devices to our telehealth center daily and on demand; (2) nurse case managers telephoned patients or caregivers daily and on demand for communication and health promotion; (3) full-time nurse case managers and cardiologists were in charge of patient care 24 hours a day; and (4) long-term medications and management were discussed with the patients’ primary care physicians after any acute event. The telehealth program bridged acute care and home care and emphasized education, prevention, and early detection of clinical deterioration.

### Data Collection

We obtained all demographic and clinical data, including the prescription of antihypertensive agents, from the electronic database of the hospital. Chronic disease diagnosis was based on the electronic database. Blood pressure and other biometric parameters were measured at home by patients or their caregivers, and data were instantaneously transmitted to the hospital server for storage and analysis. In general, patients and caregivers were advised to take measurements twice daily, with the first measurement being in the morning before eating or taking any medications and the second being in the evening. However, each patient chose their own time of day and time interval to measure their blood pressure based on their own habits. The specific model of sphygmomanometer used in this study was the AViTA BPM65ZB (AViTA Corporation, New Taipei City, Taiwan), which is an electronic digital upper arm blood pressure monitor. We obtained hourly meteorological data for Taipei (temperature, relative humidity, and wind speed) for the same time period as the study from the Central Weather Bureau, Taiwan.

### Statistical Analysis

We performed statistical analyses using R version 3.4.2 software (R Foundation). For the statistical tests, we considered a 2-sided *P* ≤.05 to be statistically significant. The distributional properties of continuous variables are expressed as mean (standard deviation), and categorical variables are presented as frequency and percentage. In the univariate analysis, we examined the differences in the distributions of continuous variables and categorical variables between male and female participants using Wilcoxon rank sum tests and Fisher exact tests, respectively.

We conducted the multivariate analysis by fitting multiple linear regression models to estimate the adjusted effects of age, sex, comorbidities, heart rate, antihypertensive drugs, seasons, meteorological factors (see below), and other predictors on 3 continuous outcomes: (1) mean blood pressure (MBP), (2) systolic blood pressure (SBP), and (3) diastolic blood pressure (DBP). Since antihypertensive drug use and meteorological factors varied over time, we defined and included the following 2 groups of time-dependent covariates in our linear regression analyses:

- Antihypertensive drugs: of the 6 frequently used categories of antihypertensive drugs (angiotensin converting enzyme inhibitors [ACEIs], angiotensin receptor blockers [ARBs], calcium channel blockers [CCBs], alpha-blockers [ABs], beta-blockers [BBs], and diuretics), we evaluated the antihypertensive drug categories and the number of categories of antihypertensive drugs used on the day of blood pressure measurement.
- Meteorological factors: we considered hourly averaged temperature, relative humidity, and wind speed within the hour of blood pressure measurement; daily highest and lowest temperatures; highest and lowest temperatures in the 12 hours prior to blood pressure measurement; highest and lowest temperatures in the 24 hours prior to blood pressure measurement; difference between the highest and lowest daily temperature; difference between the highest and lowest temperature in the 12 hours prior to blood pressure measurement; and difference between the highest and lowest temperature in the 24 hours prior to blood pressure measurement.

More statistical details can be found in Multimedia Appendix 1.

## Results

### Patient Population and Demographics

From January 2009 to December 2013, a total of 253 patients who participated in the Telehealth Care Program of National Taiwan University Hospital who had complete demographic and clinical data were enrolled in the study. We collected a total of 110,715 blood pressure measurements in the database during the study period. The demographic and clinical data are summarized in Table 1 (per patient) and Table 2 (per measurement). Briefly, the mean patient age was 70.28 (SD 13.79) years, and 66.0% (167/253) of patients were male. Hypertension was diagnosed in 61.7% (156/253) of patients, diabetes mellitus in 32.8% (83/253), heart failure in 30.4% (77/253), prior stroke in 14.2% (36/263), and CAD in 57.3% (145/253).

### Univariate Analysis

Table 1 details the demographic and clinical characteristics of the 253 patients. The distributional parameters of most variables did not differ statistically between male (n=167) and female (n=86) patients except that male patients had a younger mean age (68.73, SD 13.54 years vs 73.31, SD 13.84 years; *P*=.003), higher proportions of CAD without prior MI (72/167, 43.1% vs 32/86, 37.2%; *P*=.006) and CAD with prior MI (24/167, 20.4% vs 7/86, 8.1%; *P*=.006), and a lower proportion with cancer (17/167, 10.2% vs 18/86, 20.9%; *P*=.02). Table 2 details the demographic and clinical characteristics of 110,715 observations from the 253 patients. On average, each patient provided 437.61 repeated blood pressure measurements (male: 452.56; female: 408.57) during the study period. We recorded and counted daily use of any of the following 6 categories of antihypertensive drugs: ACEIs, ARBs, CCBs, ABs, BBs, and diuretics. In the statistical analysis, we considered the number of categories of antihypertensive drugs and the specific categories of antihypertensive drugs used on the day of blood pressure measurement. The distributional parameters of most of the variables differed statistically between male (n=75,578) and female (n=35,137) patients for 11 of the 12 meteorological factors. Given the large number of observations (110,715), the statistical power was sufficiently high to find relatively small differences between male and female patients on the statistical tests.

### Meteorological Data

The mean hourly averaged temperature was 23.43 (SD 5.69) °C, and the mean relative humidity was 72.28 (SD 9.95) %. The mean wind speed was 2.47 (SD 1.60) m/s. The mean temperature differences in the past 12 and 24 hours were 3.71 (SD 2.32) °C and 5.72 (2.60) °C, respectively (Table 2).

### Antihypertensive Agents

The most frequently used antihypertensive agent class was diuretics (45,526/110,715, 41.12%), followed by ARBs (41,465/110,715, 37.45%), CCBs (29,923/110,715, 27.03%), and BBs (23,107/110,715, 20.87%; Table 2).

### Multivariate Analysis

**Figure 1:**
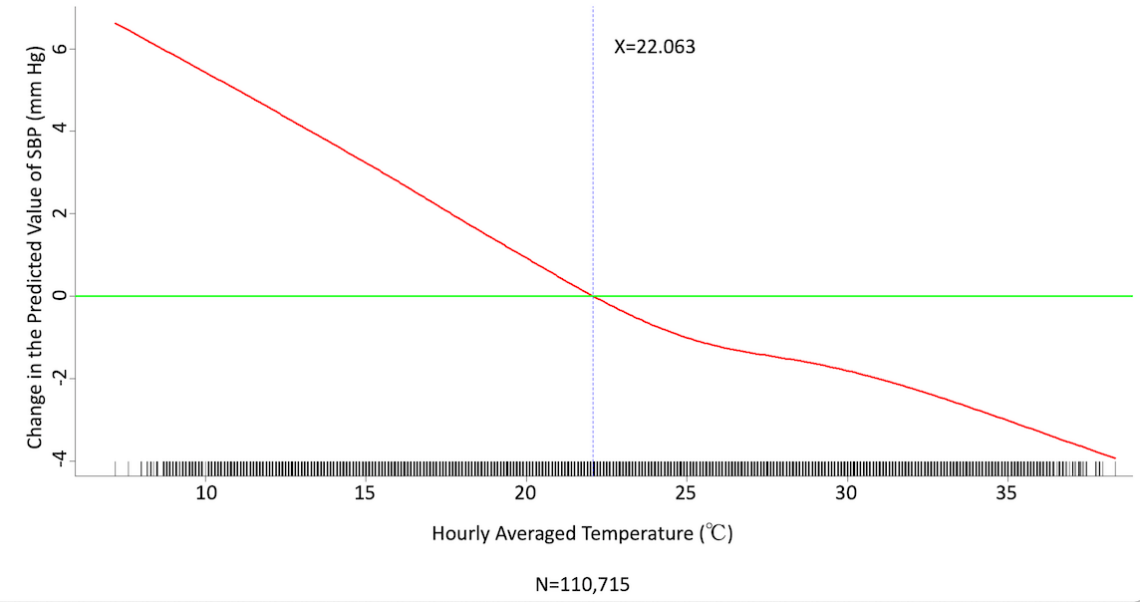
Generalized additive model plot showing the relationship between systolic blood pressure (SBP) and the hourly averaged temperature.

**Figure 2:**
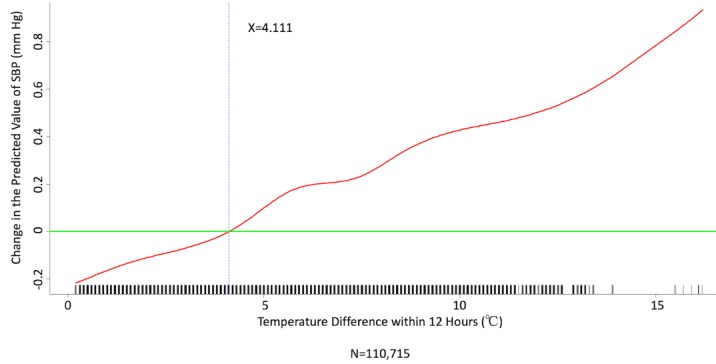
Generalized additive model plot showing the relationship between systolic blood pressure (SBP) and the temperature difference between maximum and minimum in the 12 hours prior to blood pressure measurement.

**Figure 3:**
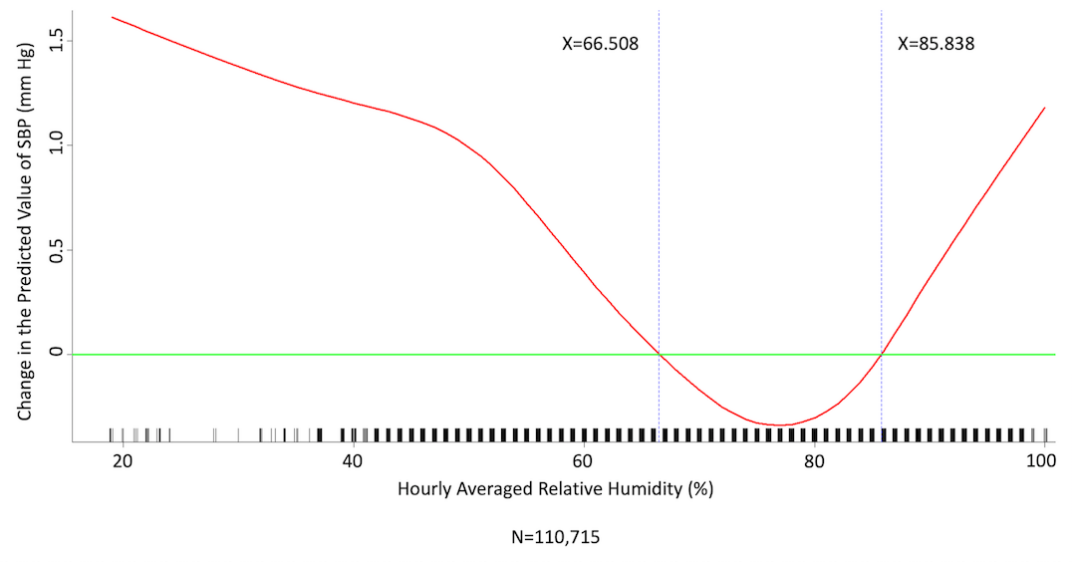
Generalized additive model plot showing the relationship between systolic blood pressure (SBP) and the hourly averaged relative humidity.

**Figure 4:**
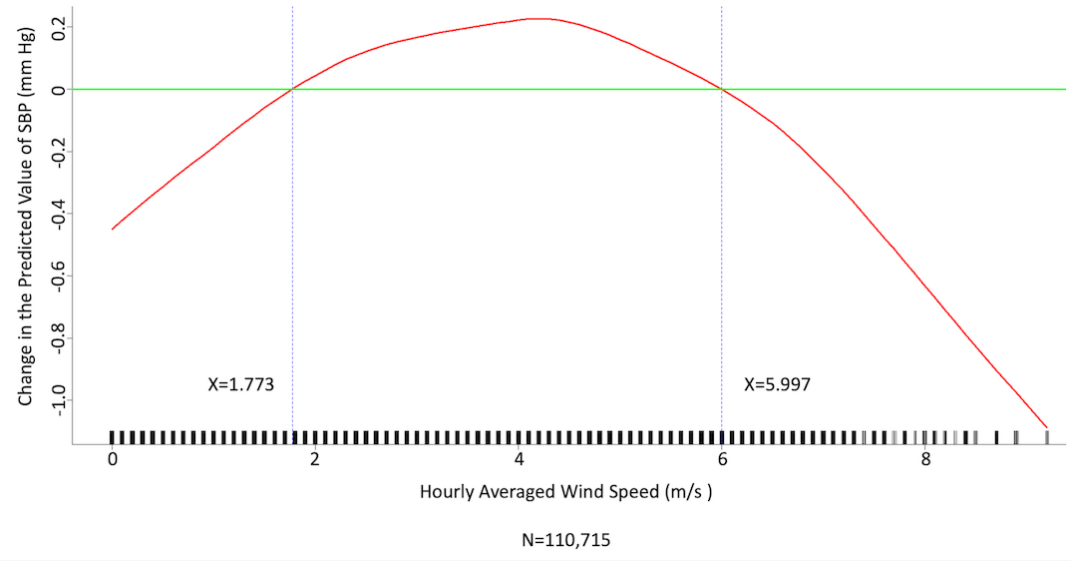
Generalized additive model plot showing the relationship between systolic blood pressure (SBP) and the hourly averaged wind speed.

**Figure 5:**
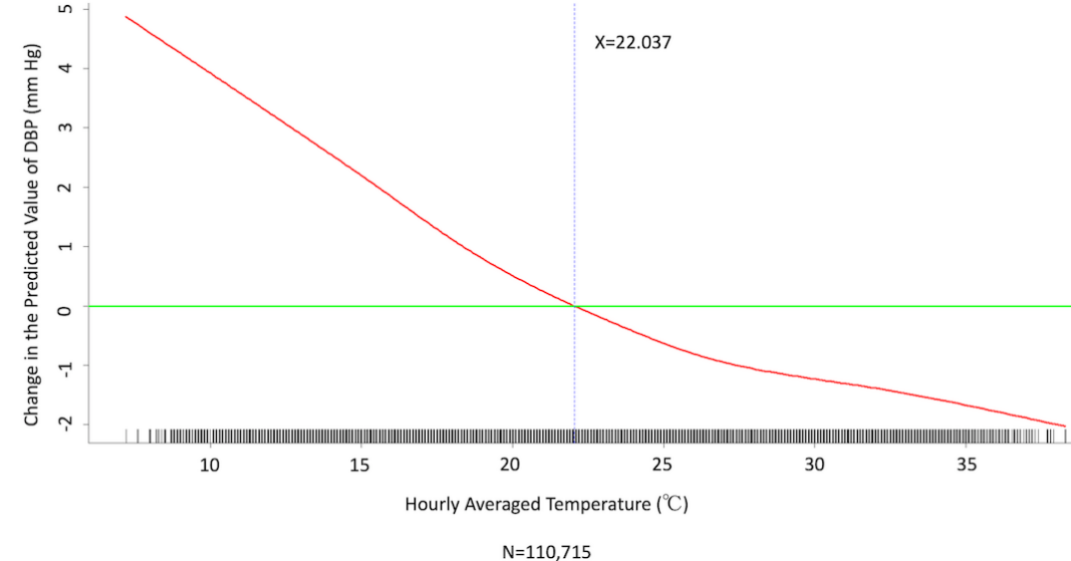
Generalized additive model plot showing the relationship between diastolic blood pressure (DBP) and the hourly averaged temperature.

**Figure 6:**
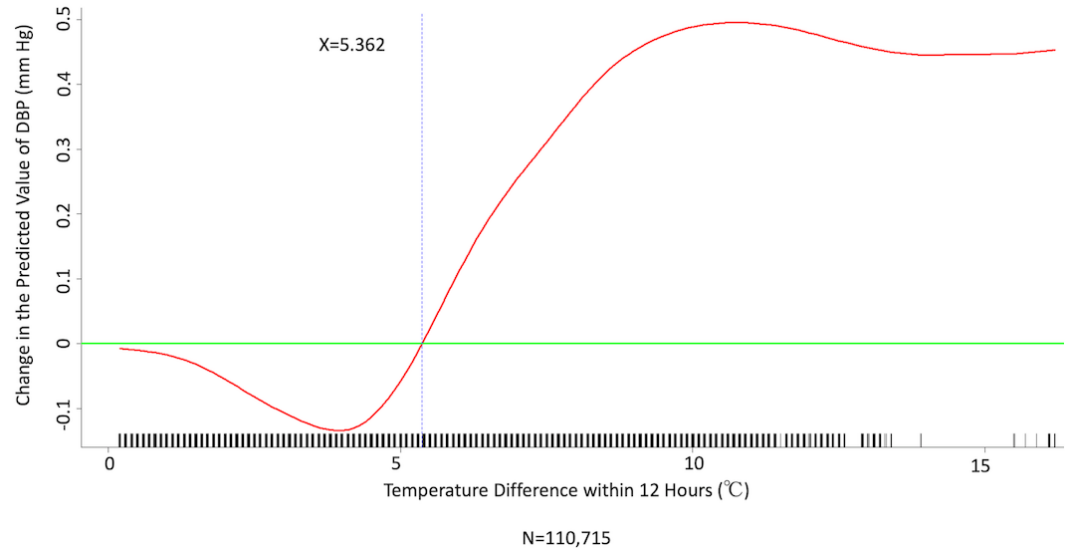
Generalized additive model plot showing the relationship between diastolic blood pressure (DBP) and the temperature difference between maximum and minimum in the 12 hours prior to blood pressure measurement.

**Figure 7:**
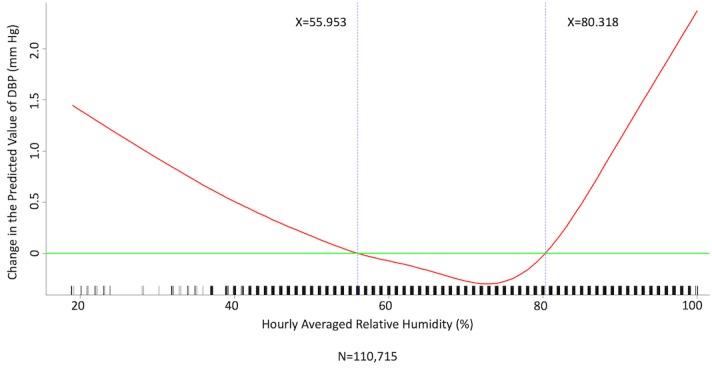
Generalized additive model plot showing the relationship between diastolic blood pressure (DBP) and the hourly averaged relative humidity.

**Figure 8:**
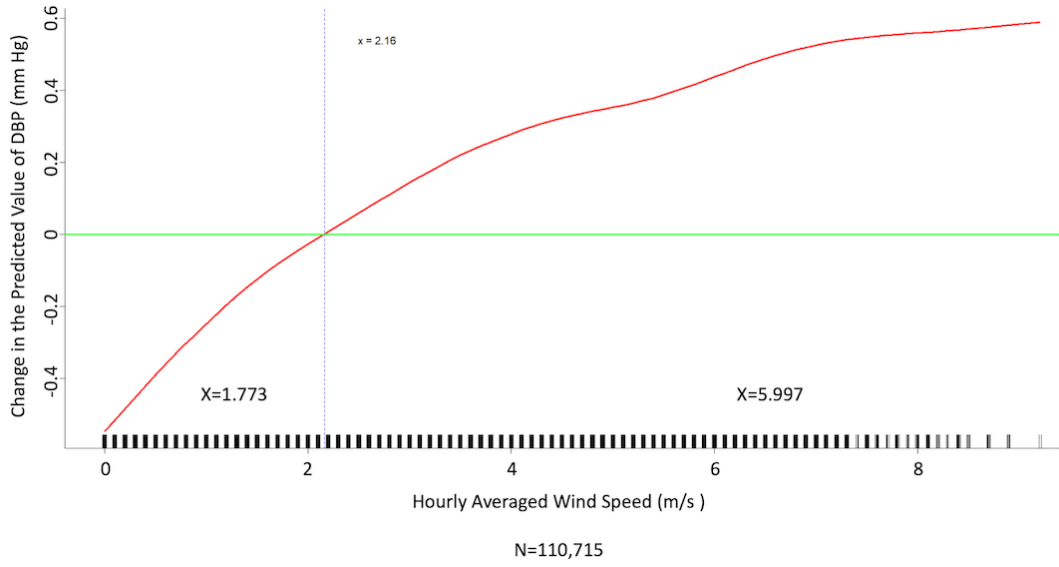
Generalized additive model plot showing the relationship between diastolic blood pressure (DBP) and the hourly averaged wind speed.

We conducted multivariate analysis by fitting multiple linear regression models to estimate the adjusted effects of age, sex, hypertension, diabetes mellitus, cancer, atrial fibrillation, congestive heart failure, CAD, prior MI, prior stroke, peripheral artery disease, heart rate, the 6 categories of antihypertensive drugs, seasons, the 12 meteorological factors, and other potential predictors on 3 continuous outcomes: (1) SBP, (2) DBP, and (3) MBP. Since patients’ use of antihypertensive drugs and meteorological factors varied over time, we defined and computed both as time-dependent covariates in these 3 regression analyses. Tables 3 and 4, and Multimedia Appendix 2 display the 3 fitted multiple final linear regression models of mean SBP (mm Hg), DBP (mm Hg), and MBP (mm Hg), respectively. As Figures 1-4 show, we determined the cutoff point(s) for discretizing continuous covariates with nonlinear effects on mean SBP (mm Hg) objectively using the corresponding generalized additive model plots during the stepwise variable selection procedure. We applied the same approach to regression analyses of DBP (mm Hg) and MBP (mm Hg), and the generalized additive model plots are shown in Figures 5-8 and Multimedia Appendix 3, respectively.

**Table 1:** Demographic and clinical characteristics of the 253 patients.

Characteristics		Male	Female	All patients	*P* value^a^
Sample size, n (%)		167 (66.0)	86 (34.0)	253 (100)	N/A^b^
Age (years), mean (SD)		68.73 (13.54)	73.31 (13.84)	70.28 (13.79)	.003
**Hypertension, n (%)**					.34
	No	68 (40.7)	29 (33.7)	97 (38.3)	
	Yes	99 (59.3)	57 (66.3)	156 (61.7)	
**Diabetes mellitus, n (%)**					.89
	No	113 (67.7)	57 (66.3)	170 (67.2)	
	Yes	54 (32.3)	29 (33.7)	83 (32.8)	
**Atrial fibrillation, n (%)**					.19
	No	138 (82.6)	65 (75.6)	203 (80.2)	
	Yes	29 (17.4)	21 (24.4)	50 (19.8)	
**Congestive heart failure, n (%)**					.47
	No	119 (71.3)	57 (66.3)	176 (69.6)	
	Yes	48 (28.7)	29 (33.7)	77 (30.4)	
**Coronary artery disease (CAD) and myocardial infarction (MI), n (%)**					.006
	No CAD	61 (36.5)	47 (54.7)	108 (42.7)	
	CAD without prior MI	72 (43.1)	32 (37.2)	104 (41.1)	
	CAD with prior MI	34 (20.4)	7 (8.1)	41 (16.2)	
**Prior stroke, n (%)**					.57
	No	145 (86.8)	72 (83.7)	217 (85.8)	
	Yes	22 (13.2)	14 (16.3)	36 (14.2)	
**Peripheral artery disease, n (%)**					.47
	No	152 (91.0)	81 (94.2)	233 (92.1)	
	Yes	15 (9.0)	5 (5.8)	20 (7.9)	
**Cancer, n (%)**					.02
	No	150 (89.8)	68 (79.1)	218 (86.2)	
	Yes	17 (10.2)	18 (20.9)	35 (13.8)	

^a^Calculated using the Wilcoxon rank sum test for continuous variables and the Fisher exact test for categorical variables.

^b^N/A: not applicable.

Multimedia Appendix 2 presents the fitted multiple linear regression model of mean MBP (mm Hg) as a linear equation for outcome prediction. Hourly averaged temperature (°C) within the hour of blood pressure measurement had a negative relationship with the mean value of MBP with a slope of −0.55 mm Hg/°C (Multimedia Appendix 3), but this negative slope was modified to some extent by the covariates hypertension, diabetes, ACEIs, ARBs, CCBs, and diuretics, which are the so-called effect modifiers in the epidemiological and statistical literature. In Tables 3 and 4, the fitted multiple linear regression models modeling mean SBP (mm Hg) and DBP (mm Hg), respectively, can be interpreted in the same manner.

### Effects of Meteorological Factors on Home Blood Pressure

Hourly averaged temperature had a linear negative effect on all 3 HBP parameters, so lower temperature resulted in higher HBP. The temperature difference between the maximum and minimum in the 12 hours prior to blood pressure measurement had a positive effect on MBP and DBP (ie, a larger temperature difference resulted in higher MBP and DBP), but the effect was not significant for SBP. Relative humidity and wind speed had nonlinear effects on HBP, as shown in the generalized additive model plots (Figures 1-4 for SBP, Figures 5-8 for DBP, and Multimedia Appendix 3 for MBP).

**Table 2:** Demographic and clinical characteristics of the 110,715 blood pressure observations from the 253 patients.

Characteristics			Male (n=167)	Female (n=86)	All patients (n=253)	*P* value^a^	
Observations, n (%)			75,578 (68.26)	35,137 (31.74)	110,715 (100)	N/A^b^	
Age (years), mean (SD)			72.19 (12.87)	79.82 (10.23)	74.61 (12.61)	<.001	
Mean blood pressure (mm Hg), mean (SD)			106.33 (12.95)	108.06 (13.57)	106.88 (13.17)	<.001	
Systolic blood pressure (mm Hg), mean (SD)			125.08 (16.43)	127.73 (16.76)	125.92 (16.58)	<.001	
Diastolic blood pressure (mm Hg), mean (SD)			68.83 (11.85)	68.72 (11.65)	68.79 (11.79)	.002	
Heart rate (beats/min), mean (SD)			68.73 (12.43)	73.32 (11.92)	70.18 (12.46)	<.001	
**Antihypertensive drugs used on the day of blood pressure measurement**							
	Number of categories of used antihypertensive drugs, mean (SD)		1.33 (1.08)	1.50 (1.11)	1.39 (1.09)	<.001	
	**1. Angiotensin converting enzyme inhibitors, n (%)**						<.001
		No	73,250 (96.92)	33,922 (96.54)	107,172 (96.80)		
		Yes	2,328 (3.08)	1,215 (3.46)	3,543 (3.20)		
	**2. Angiotensin receptor blocker, n (%)**						<.001
		No	49,161 (65.05)	20,089 (57.17)	69,250 (62.55)		
		Yes	26,417 (34.95)	15,048 (42.83)	41,465 (37.45)		
	**3. Calcium channel blockers, n (%)**						<.001
		No	56,953 (75.36)	23,839 (67.85)	80,792 (72.97)		
		Yes	18,625 (24.64)	11,298 (32.15)	29,923 (27.03)		
	**4. Alpha-blockers, n (%)**						<.001
		No	66,043 (87.38)	34,707 (98.78)	100,750 (91.00)		
		Yes	9,535 (12.62)	430 (1.22)	9,965 (9.00)		
	**5. Beta-blockers, n (%)**						.005
		No	59,982 (79.36)	27,626 (78.62)	87,608 (79.13)		
		Yes	15,596 (20.64)	7,511 (21.38)	23,107 (20.87)		
	**6. Diuretics, n (%)**						<.001
		No	47,422 (62.75)	17,767 (50.57)	65,189 (58.88)		
		Yes	28,156 (37.25)	17,370 (49.44)	45,526 (41.12)		
**Season, n (%)**							<.001
	Spring		16,243 (21.49)	7,870 (22.40)	24,113 (21.78)		
	Summer		18,929 (25.05)	8,851 (25.19)	27,780 (25.09)		
	Fall		22,143 (29.30)	9,963 (28.36)	32,106 (29.00)		
	Winter		18,263 (24.16)	8,453 (24.06)	26,716 (24.13)		
**Meteorological factors, mean (SD)**							
	Hourly averaged temperature^c^ (°C)		23.45 (5.69)	23.39 (5.69)	23.43 (5.69)	.21	
	Hourly averaged relative humidity^c^ (%)		72.28 (9.96)	72.28 (9.94)	72.28 (9.95)	.95	
	Hourly averaged wind speed^c^ (m/s)		2.48 (1.60)	2.46 (1.60)	2.47 (1.60)	.09	
	Daily maximum temperature^d^ (°C)		26.60 (6.24)	26.59 (6.28)	26.59 (6.25)	.86	
	Daily minimum temperature^d^ (°C)		20.90 (5.05)	20.89 (5.09)	20.90 (5.06)	.83	
	Difference between daily maximum and daily minimum temperatures^d^ (°C)		5.70 (2.65)	5.70 (2.66)	5.70 (2.65)	.83	
	Maximum temperature in the 12 hours prior to blood pressure measurement (°C)		25.32 (6.12)	25.31 (6.15)	25.32 (6.13)	.72	
	Minimum temperature in the 12 hours prior to blood pressure measurement (°C)		21.60 (5.12)	21.62 (5.19)	21.60 (5.14)	.37	
	Difference between maximum and minimum temperatures in the 12 hours prior to blood pressure measurement (°C)		3.73 (2.32)	3.69 (2.30)	3.71 (2.32)	.04	
	Maximum temperature in the 24 hours prior to blood pressure measurement (°C)		26.73 (6.18)	26.72 (6.22)	26.73 (6.19)	.99	
	Minimum temperature in the 24 hours prior to blood pressure measurement (°C)		21.01 (5.05)	21.00 (5.09)	21.01 (5.06)	.88	
	Difference between maximum and minimum temperatures in the 24 hours prior to blood pressure measurement (°C)		5.72 (2.60)	5.72 (2.61)	5.72 (2.60)	.63	

^a^Calculated using the Wilcoxon rank sum test for continuous variables and the Fisher exact test for categorical variables.

^b^N/A: not applicable.

^c^Hourly averaged temperature, hourly averaged relative humidity, and hourly averaged wind speed were the readings within the *hour* of blood pressure measurement.

^d^Daily maximum temperature and daily minimum temperature were the readings within the *day* of blood pressure measurement.

### Effect of Temperature Modified by Antihypertensive Agents on Home Blood Pressure

Antihypertensive agents significantly affected HBP measurements and may modify the effect of ambient temperature (Tables 3 and 4, and Multimedia Appendix 2) on blood pressure to some extent. Only ARBs, CCBs, and diuretics significantly modified the effect of temperature on SBP (Table 3). Use of ARBs and CCBs alleviated the negative effect of temperature on SBP (from −0.6841 mm Hg/°C to –0.5523 mm Hg/°C for ARBs and to –0.6426 mm Hg/°C for CCBs), but use of diuretics further increased the negative effect of temperature on SBP (–2.2772 mm Hg/°C). For MBP, use of ACEIs, ARBs, and CCBs attenuated the negative effect of temperature, but use of diuretics potentiated the negative effect of temperature (Multimedia Appendix 2). For DBP, all antihypertensive agent classes increased the negative effect of temperature except for ARBs (Table 4). Therefore, only use of ARBs decreased the effect of temperature on all 3 HBP parameters.

### Effect of Temperature Modified by Diabetes Mellitus or Hypertension on Home Blood Pressure

Interestingly, in patients with DM or hypertension, the effect of ambient temperature on HBP was less pronounced. Multimedia Appendix 4 shows a conditional effect plot of temperature on SBP for 65-year-old men with a history of CAD.

**Table 3:** Multivariate analysis of predictors for systolic blood pressure (SBP) by fitting a multiple linear regression model with the stepwise variable selection method.

Covariate	Parameter estimate	Standard error	*t* value	*P* r>|*t* |
Intercept	118.7080	0.4070	291.6461	<.001
Age (years)	0.2346	0.0043	54.6593	<.001
Male	0.9285	0.1134	8.1917	<.001
CAD^a^ without MI^b^ vs no CAD	2.6639	0.1071	24.8780	<.001
CAD with MI vs no CAD	−1.4940	0.1634	−9.1437	<.001
Congestive heart failure	−4.2668	0.1154	−36.9753	<.001
Peripheral artery disease	−4.2366	0.1861	−22.7713	<.001
Cancer	3.9610	0.1377	28.7566	<.001
Hourly averaged temperature^c^ (°C)	−0.6841	0.0092	−74.0842	<.001
Hypertension × hourly averaged temperature^c^ (°C)	0.1488	0.0044	33.5195	<.001
Diabetes × hourly averaged temperature^c^ (°C)	0.1776	0.0045	39.8797	<.001
Hourly averaged relative humidity^c^ ≤66.508% or hourly averaged relative humidity^c^ >85.838%	0.7227	0.0945	7.6479	<.001
Number of categories of used antihypertensive drugs^d^	0.8770	0.1110	7.9004	<.001
ARB^d^ × hourly averaged temperature^c^ (°C)	0.1318	0.0062	21.2490	<.001
ARB^d^ × AB^d^	6.9533	0.4036	17.2274	<.001
ARB^d^ × BB^d^	2.1025	0.2311	9.0969	<.001
ARB^d^ × CCB^d^	−4.4110	0.2163	−20.3951	<.001
CCB^d^ × hourly averaged temperature^c^ (°C)	0.0415	0.0074	5.6260	<.001
CCB^d^ × AB^d^	−10.2323	0.3818	−26.8019	<.001
CCB^d^ × BB^d^	−3.4101	0.2855	−11.9423	<.001
Diuretics^d^	−1.7931	0.1632	−10.9847	<.001
Diuretics^d^ × AB^d^	6.2103	0.2903	21.3900	<.001

^a^CAD: coronary artery disease.

^b^MI: myocardial infarction.

^c^Hourly averaged temperature, hourly averaged relative humidity, and hourly averaged wind speed were the readings within the *hour* of blood pressure measurement, and thus varied over time.

^d^Daily use of any of the 6 categories of antihypertensive drugs—angiotensin converting enzyme inhibitors, angiotensin receptor blockers (ARB), calcium channel blockers (CCB), alpha-blockers (AB), beta-blockers (BB), and diuretics—were recorded and counted for all 253 patients. The listed antihypertensive drugs were those taken on the *day* of blood pressure measurement, and thus may have varied over time.

**Table 4:** Multivariate analysis of predictors for diastolic blood pressure (DBP) by fitting multiple linear regression model with the stepwise variable selection method.

Covariate	Parameter estimate	Standard error	*t* value	*P* r>|*t* |
Intercept	98.7106	0.2923	337.7143	<.001
Age (years)	−0.3451	0.0030	−116.6101	<.001
Male	−0.8468	0.0789	−10.7338	<.001
CAD^a^ without MI^b^ vs no CAD	−0.5255	0.0736	−7.1373	<.001
CAD with MI vs no CAD	−0.8580	0.1143	−7.5038	<.001
Congestive heart failure	−0.6366	0.0812	−7.8369	<.001
Stroke	3.7983	0.0888	42.7668	<.001
Peripheral artery disease	2.1644	0.1281	16.8949	<.001
Cancer	−0.5488	0.0946	−5.8035	<.001
Hourly averaged temperature^c^ (°C)	−0.2709	0.0074	−36.5555	<.001
Hypertension × hourly averaged temperature^c^ (°C)	0.1151	0.0030	37.8255	<.001
Diabetes × hourly averaged temperature^c^ (°C)	0.0631	0.0030	20.6944	<.001
Hourly averaged relative humidity^c^ ≤55.953% or hourly averaged relative humidity^c^ >80.318%	0.8000	0.0756	10.5798	<.001
Hourly averaged wind speed^c^ (m/s)	0.1549	0.0200	7.7402	<.001
Temperature difference between the maximum and minimum in the 12 hours prior to blood pressure measurement >5.362°C	0.3597	0.0798	4.5053	<.001
AB^d^ × hourly averaged temperature^c^ (°C)	−0.0709	0.0086	−8.2254	<.001
ACEI^d^ × hourly averaged temperature^c^ (°C)	−0.0439	0.0097	−4.5262	<.001
ARB^d^ × hourly averaged temperature^c^ (°C)	0.0740	0.0046	16.2087	<.001
ARB^d^ × AB^d^	3.7984	0.3048	12.4603	<.001
ARB^d^ × BB^d^	6.5456	0.1632	40.0987	<.001
ARB^d^ × CCB^d^	−4.0171	0.1504	−26.7066	<.001
ARB^d^ × diuretics^d^	−3.4974	0.1401	−24.9670	<.001
BB^d^ × hourly averaged temperature^c^ (°C)	−0.0346	0.0044	−7.9548	<.001
CCB^d^ × hourly averaged temperature^c^ (°C)	−0.0141	0.0053	−2.6683	.008
CCB^d^ × AB^d^	2.0324	0.2695	7.5417	<.001
CCB^d^ × ACEI^d^	−11.8642	0.8327	−14.2472	<.001
CCB^d^ × BB^d^	−0.6426	0.1978	−3.2479	.001
CCB^d^ × diuretics^d^	3.0658	0.1502	20.4120	<.001
Diuretics^d^ × hourly averaged temperature ^c^ (°C)	−0.0329	0.0044	−7.4992	<.001
Diuretics^d^ × AB ^d^	−5.6251	0.2490	−22.5946	<.001
Diuretics^d^ × ACEI ^d^	2.8185	0.3888	7.2483	<.001

^a^CAD: coronary artery disease.

^b^MI: myocardial infarction.

^c^Hourly averaged temperature, hourly averaged relative humidity, and hourly averaged wind speed were the readings within the *hour* of blood pressure measurement, and thus varied over time.

^d^Daily use of any of the 6 categories of antihypertensive drugs—angiotensin converting enzyme inhibitors (ACEI), angiotensin receptor blockers (ARB), calcium channel blockers (CCB), alpha-blockers (AB), beta-blockers (BB), and diuretics—were recorded and counted for all 253 patients. The listed antihypertensive drugs were those taken on the *day* of blood pressure measurement, and thus may have varied over time.

## Discussion

### Effect of Meteorological Factors on Home Blood Pressure

Both extremely high and low temperatures have been shown to increase mortality [2], but the underlying mechanism is not well understood. Seasonal variations in blood pressure have been well reported, and it was assumed to be one of the possible underlying mechanisms contributing to low-temperature–related mortality increases. However, most previous studies have been population or cross-sectional studies.

Hintsala et al reported that even short-term cold exposure increased central aortic blood pressure [23], and the underlying mechanism may be endothelial-dependent vasodilation [24]. HBP was recently found to be a more powerful predictor than office blood pressure of future cardiovascular events [14). To the best of our knowledge, our study is the first to investigate the relationships among demographics, meteorological factors, medications, and HBP at the patient level. The study had a few important findings. HBP was significantly affected by meteorological factors. Of the meteorological factors we investigated, ambient temperature (hourly averaged temperature) was the most important and had a linear inverse relationship with all 3 of the HBP parameters. Kimura et al [15] and Imai et al [16] reported seasonal HBP variations, but their participants were healthy normotensive elderly individuals and their studies comprised limited patient numbers [15,16]. Iwahori et al [17] also recently confirmed seasonal variations in HBP by using a nationwide Web-based databank in Japan, although effects on individual participants based on repeated measurement could not be investigated, as the study was a population study. Our study confirmed that temperature has an inverse linear effect on HBP in patients with chronic cardiovascular diseases. Moreover, the results showed that both absolute temperature and temperature difference affected HBP. Relative humidity and wind speed also had significant effects on HBP, and the relationships appear to be nonlinear.

### Effect of Temperature Modified by Antihypertensive Agents on Home Blood Pressure

In this study, patients with a diagnosis of either diabetes mellitus or hypertension appeared to be more resistant to the effect of temperature on HBP. The true underlying mechanism remains unknown, but medications appear to play a role. Chen el al [10] reported that benazepril attenuated temperature-mediated blood pressure variations. We included the 6 most frequently used antihypertensive agent classes in the multivariate analysis and found that antihypertensive agents significantly modified the effect of temperature on HBP to some extent. However, the effect of each individual drug class was variable. Of the 6 classes of antihypertensive agents, ARBs appeared to have the most favorable outcome, as use of ARBs attenuated the negative effect of temperature on all 3 HBP parameters. On the other hand, use of diuretics appeared to potentiate the negative effect of temperature on HBP, as the slope increased for all HBP parameters. Although diuretics (thiazides) are still among the most frequently used antihypertensive agents in practice and are suggested by the current guidelines as one of the first-line agents [13], this study showed that patients taking diuretics were more sensitive to temperature changes. In this regard, ARBs may be the class of choice for patients whose blood pressure is more sensitive to ambient temperature.

The fact that ACEIs had no significant influence on HBP, in contrast to ARBs, was somewhat surprising. However, the number of patients taking ACEIs was very small, so the analysis in this study may not have had sufficient power to detect the true effect of ACEIs. On the other hand, the proportion of patients taking ARBs and diuretics was the largest of the various classes, and this may have increased the power of the analysis to find an effect.

### Impact on Health

As the final multiple linear regression models shown in Tables 3 and 4 suggest, changes to any covariate in the regression model had positive and negative effects on mean SBP and DBP. While the values of the other covariates were held fixed, mean SBP increased by 0.6841 mm Hg (Table 3) and mean DBP increased by 0.2709 mm Hg (Table 4) within an hour due to a 1°C decrease in the hourly averaged environmental temperature. According to a meta-analysis, every 4-mm Hg reduction in SBP and 2-mm Hg reduction in DBP is sufficient to significantly reduce cardiovascular events [25]. The effect of temperature on HBP may thus have a huge impact on health. Additional longer-term outcome studies are warranted to confirm this phenomenon.

### Study Limitations

This study was a retrospective registry with a relatively small number of patients. The patients all had chronic cardiovascular diseases with excellent adherence as they participated in a telehealth care program; thus, the study results should be extrapolated to other patient populations with caution. The ambient temperature was outdoor temperature in this study, and thus may have underestimated the true effect of indoor temperature. Previous studies have shown that both indoor and outdoor temperatures are inversely related to blood pressure, and there is a stronger association with indoor temperature than with outdoor temperature [8,26-29]. The true effect of indoor temperature on HBP should thus be more prominent.

We did not include medications other than antihypertensive agents, such as oral antidiabetic drugs and statins, in this analysis. As a result, whether these drugs also modify the effect of temperature on HBP is unknown. We also did not include other environmental factors such as air pollutants in this study. Moreover, although the patients and caregivers were advised to take measurements twice daily, patients and their caregivers chose their own time of day and time intervals to measure blood pressure. There may thus be heterogeneity across patients regarding measurement behavior and potential confounding effects.

Since the repeated measurements of SBP and DBP on the same patient were probably correlated, we could have applied the generalized estimating equations method to obtain robust estimates of standard errors for the estimated regression coefficients of the multiple linear regression models of SBP and DBP. However, we did not do so because the cluster sizes were too big (ie, many repeated measurements on some patients).

### Conclusions

Short-term exposure to low ambient temperature significantly increased HBP in patients with chronic cardiovascular diseases, and antihypertensive agents could have modified this effect.

## Appendix

Multimedia Appendix 1 More statistical details.

Multimedia Appendix 2 Multivariate analysis of predictors for mean blood pressure.

Multimedia Appendix 3 Generalized additive model plots of the relationship between mean blood pressure and meteorological factors.

Multimedia Appendix 4 Conditional effect plot of temperature on value of systolic blood pressure for 65-year-old men with history of coronary artery disease.

## Abbreviations

AB: alpha-blocker
ACEI: angiotensin converting enzyme inhibiter
ARB: angiotensin receptor blocker
BB: beta-blocker
CAD: coronary artery disease
CCB: calcium channel blocker
DBP: diastolic blood pressure
HBP: home blood pressure
MBP: mean blood pressure
MI: myocardial infarction
SBP: systolic blood pressure
