# Supplementary material for: Assessment of the Relationship Between Ambient Temperature and Home Blood Pressure in Patients From a Web-Based Synchronous Telehealth Care Program: Retrospective Study
Source: J Med Internet Res. 2019 Mar 4;21(3):e12369. doi: 10.2196/12369 (PMC6421515; doi:10.2196/12369)
Supplement: Multimedia Appendix 1 [file jmir_v21i3e12369_app4.pdf]

Conditional effect plot of temperature on systolic blood pressure for 65-year-old men with a history of coronary artery disease. The negative effect of ambient temperature was smaller for patients with diabetes or hypertension than it was for patients without either of them.

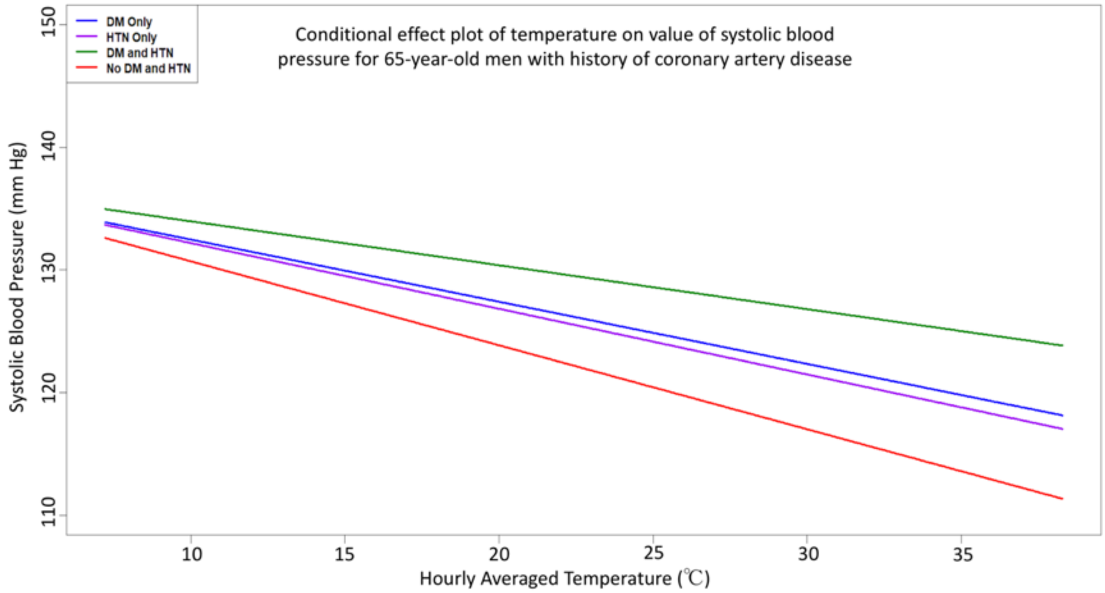

Conditional effect plot of temperature on value of systolic blood pressure for 65-year-old men with history of coronary artery disease.
