# Supplementary material for: Assessment of the Relationship Between Ambient Temperature and Home Blood Pressure in Patients From a Web-Based Synchronous Telehealth Care Program: Retrospective Study
Source: J Med Internet Res. 2019 Mar 4;21(3):e12369. doi: 10.2196/12369 (PMC6421515; doi:10.2196/12369)
Supplement: Multimedia Appendix 2 [file jmir_v21i3e12369_app1.pdf]

More Statistical Details.

Univariate and multivariate analyses. The goal of regression analysis was to find one or a few parsimonious regression models that fitted the observed data well for effect estimation and/or outcome prediction. To ensure a good quality of analysis, the model-fitting techniques for (1) variable selection, (2) goodness-of-fit (GOF) assessment, and (3) regression diagnostics and remedies were used in our linear regression analyses. All the univariate significant and non-significant relevant covariates (listed in Table 1) and some of their interaction terms (or moderators) were put on the variable list to be selected. The significance levels for entry (SLE) and for stay (SLS) were set to 0.15 for being conservative. Then, with the aid of substantive knowledge, the best candidate final linear regression model was identified manually by dropping the covariates with p value > 0.05 one at a time until all regression coefficients were significantly different from 0. Any discrepancy between the results of univariate analysis and multivariate analysis was likely due to the confounding effects of uncontrolled covariates in univariate analysis or the masking effects of intermediate variables (or mediators) in multivariate analysis. The GOF measure, coefficient of determination R2, was examined to assess the GOF of the fitted linear regression model. Technically, the R2 statistic ($0 \le R2 \le 1$) for linear regression model equals the square of the Pearson correlation between the observed and

predicted response values and it indicates how much of the response variability is explained by the covariates included in the linear regression model. Simple and multiple generalized additive models (GAMs) were fitted to detect nonlinear effects of continuous covariates and identify appropriate cut-off point(s) for discretizing continuous covariates, if necessary, during the stepwise variable selection procedure. Finally, the statistical tools of regression diagnostics for residual analysis, detection of influential cases, and check of multicollinearity were applied to discover any model or data problems. The values of variance inflating factor (VIF) ≥ 10 in continuous covariates or ≥ 2.5 in categorical covariates indicate the occurrence of the multicollinearity problem among some of the covariates in the fitted linear regression model.

Stepwise variable selection. The stepwise variable selection procedure (with iterations between the forward and backward steps) was applied to obtain the best candidate final linear regression model using the My.stepwise.lm() function of the My.stepwise package in R (Hu, 2017). Computationally, the vgam() function (with the default values of smoothing parameters) of the VGAM package (Yee and Wild, 1996; Yee, 2015, 2017) was used to fit GAMs for our continuous responses in R.

Linear equation for outcome prediction. In Additional Table 1, the fitted multiple linear

regression model for modeling the mean value of MBP (mm Hg) can be written as the following linear equation for outcome prediction:

Mean of MBP (mm Hg) = 114.93 − 2.90 × **I**(Age ≤ 58.0 years) + 0.83 × **I**(58.0 years < Age ≤ 73.8 years) + 0.49 × **I**(Male) + 1.26 × **I**(CAD without MI) − 1.02 × **I**(CAD with MI) − 3.02 × **I**(CHF) + 1.99 × **I**(CVA) − 1.83 × **I**(PAOD) + 2.42 × **I**(Cancer) − 0.55 × Hourly averaged temperature (˚C) + 0.13 × **I**(Hypertension) × Hourly averaged temperature (˚C) + 0.15 × **I**(Diabetes) × Hourly averaged temperature (˚C) + 0.06 × Temperature difference between maximum and minimum in 12 hours prior to blood pressure measurement (˚C) + 0.58 × **I**(Hourly averaged relative humidity ≤ 61.432% or Hourly averaged relative humidity > 81.51%) + 0.40 × **I**(2.046 m/s < Hourly averaged wind speed[1] ≤ 7.313 m/s) + 0.23 × Number of categories of used antihypertensive drugs + 0.03 × **I**(ACEI) × Hourly averaged temperature (˚C) + 0.14 × **I**(ARB) × Hourly averaged temperature (˚C) + 6.66 × **I**(ARB) × **I**(AB)+ 3.69 × **I**(ARB) × **I**(BB) − 4.65 × **I**(ARB) × **I**(CCB) − 1.77 × **I**(ARB) × **I**(Diuretics) + 0.04 × **I**(CCB) × Hourly averaged temperature (˚C) − 5.83 × **I**(CCB) × I(AB) − 6.79 × **I**(CCB) × **I**(ACEI) − 2.11 × **I**(CCB) × I(BB) + 1.59 × **I**(CCB) × **I**(Diuretics) − 0.04 × **I**(Diuretics) × Hourly averaged temperature (˚C) + 2.21 × **I**(Diuretics) × **I**(AB) + 1.42 × **I**(Diuretics) × **I**(ACEI)

where the cross sign “×” in the interaction terms just means numeric multiplications and the

indicator function **I**(•) = 1 if the condition specified in the parentheses was met and 0 otherwise. Clearly, the variables with positive values of regression coefficient estimates increased the mean value of MBP, whereas the ones with negative values of regression coefficient estimates decrease it. In particular, to see the effect of hourly averaged temperature (˚C) within the hour of blood pressure measurement on the mean value of MBP, we can group all the terms with it together as below:

− 0.55 × Hourly averaged temperature (˚C) + 0.13 × **I**(Hypertension) × Hourly averaged temperature (˚C) + 0.15 × **I**(Diabetes) × Hourly averaged temperature (˚C) + 0.03 × **I**(ACEI) × Hourly averaged temperature (˚C) + 0.14 × **I**(ARB) × Hourly averaged temperature (˚C) + 0.04 × **I**(CCB) × Hourly averaged temperature (˚C) − 0.04 × **I**(Diuretics) × Hourly averaged temperature (˚C)

which equals

[− 0.55 + 0.13 × **I**(Hypertension) + 0.15 × **I**(Diabetes) + 0.03 × **I**(ACEI) + 0.14 × **I**(ARB) + 0.04 × **I**(CCB) − 0.04 × **I**(Diuretics)] × Hourly averaged temperature (˚C).

By doing so, we can see more easily that hourly averaged temperature (˚C) within the hour of blood pressure measurement had a negative effect on the mean value of MBP with the slope of −0.55 mm Hg/˚C (Figure 2), but this negative slope could be modified to some extent by the

covariate values of **I**(Hypertension), **I**(Diabetes), **I**(ACEI), **I**(ARB), **I**(CCB), and **I**(Diuretics) respectively, which were the so-called "effect modifiers" in epidemiological and statistical literature. In Tables 3 and 4, the fitted multiple linear regression models for modeling the mean values of SBP (mm Hg) and DBP (mm Hg) respectively can be interpreted in the same way.
