# Supplementary material for: Assessment of the Relationship Between Ambient Temperature and Home Blood Pressure in Patients From a Web-Based Synchronous Telehealth Care Program: Retrospective Study
Source: J Med Internet Res. 2019 Mar 4;21(3):e12369. doi: 10.2196/12369 (PMC6421515; doi:10.2196/12369)
Supplement: Multimedia Appendix 3 [file jmir_v21i3e12369_app2.pdf]

Multivariate analysis of predictors for mean blood pressure (MBP) by fitting a multiple linear regression model with the stepwise variable selection method

Covariate	Parameter Estimate	Standard Error	*t* Value	Pr > |*t*|
Intercept	114.9281	0.2331	493.0257	<0.0001
Age ≤ 57.981 years	−2.8962	0.1361	−21.2771	<0.0001
57.981 years < Age ≤ 73.824 years	0.8255	0.0993	8.3129	<0.0001
Male	0.4930	0.0941	5.2377	<0.0001
CAD without MI vs. No CAD	1.2647	0.0878	14.3996	<0.0001
CAD with MI vs. No CAD	−1.0178	0.1357	−7.4988	<0.0001
CHF	−3.0234	0.0960	−31.5033	<0.0001
CVA	1.9922	0.1060	18.7872	<0.0001
PAD	−1.8266	0.1511	−12.0916	<0.0001
Cancer	2.4246	0.1114	21.7619	<0.0001
Hourly averaged temperature[1] (˚C)	−0.5492	0.0090	−61.0805	<0.0001
Hypertension × Hourly averaged temperature[1] (˚C)	0.1286	0.0036	35.3920	<0.0001
Diabetes × Hourly averaged temperature[1] (˚C)	0.1452	0.0036	40.0787	<0.0001
Temperature difference between the maximum and minimum in the 12 hours prior to blood pressure measurement (˚C)	0.0612	0.0182	3.3678	0.0008
Hourly averaged relative humidity[1] ≤ 61.432% or Hourly averaged relative humidity[1] > 81.51%	0.5791	0.0796	7.2714	<0.0001
2.046 m/s < Hourly averaged wind speed[1] ≤ 7.313 m/s	0.4034	0.0761	5.2978	<0.0001
Number of categories of used antihypertensive drugs[2]	0.2263	0.1014	2.2328	0.0256
ACEI[2] × Hourly averaged temperature[1] (˚C)	0.0345	0.0124	2.7798	0.0054
ARB[2] × Hourly averaged temperature[1] (˚C)	0.1404	0.0060	23.5586	<0.0001
ARB[2] × AB[2]	6.6573	0.3368	19.7689	<0.0001
ARB[2] × BB[2]	3.6949	0.1902	19.4305	<0.0001
ARB[2] × CCB[2]	−4.6539	0.1801	−25.8340	<0.0001
ARB[2] × Diuretics[2]	−1.7671	0.1688	−10.4674	<0.0001

$CCB^{2} \times$ Hourly averaged temperature[1] (˚C)	0.0432	0.0070	6.2002	<0.0001
$CCB^{2} \times AB^{2}$	−5.8290	0.3102	−18.7928	<0.0001
$CCB^{2} \times ACEI^{2}$	−6.7877	0.9862	−6.8827	<0.0001
$CCB^{2} \times BB^{2}$	−2.1052	0.2351	−8.9546	<0.0001
$CCB^{2} \times Diuretics^{2}$	1.5916	0.1784	8.9233	<0.0001
$Diuretics^{2} \times$ Hourly averaged temperature[1] (˚C)	−0.0433	0.0060	−7.2314	<0.0001
$Diuretics^{2} \times AB^{2}$	2.2120	0.2514	8.7989	<0.0001
$Diuretics^{2} \times ACEI^{2}$	1.4163	0.4620	3.0656	0.0022

[1] Hourly averaged temperature, hourly averaged relative humidity, and hourly averaged wind speed were the readings within the *hour* of blood pressure measurement so vary over time.

[2] Daily use of any of the following six categories of antihypertensive drugs, angiotensin converting enzyme inhibitors (ACEI), angiotensin receptor blockers (ARB), calcium-channel blockers (CCB), alpha-blockers (AB), beta-blockers (BB), and diuretics, were recorded and counted for all 253 patients. The listed antihypertensive drugs were those taken on the *day* of blood pressure measurement, so may vary over time.
