# Supplementary material for: Assessment of the Relationship Between Ambient Temperature and Home Blood Pressure in Patients From a Web-Based Synchronous Telehealth Care Program: Retrospective Study
Source: J Med Internet Res. 2019 Mar 4;21(3):e12369. doi: 10.2196/12369 (PMC6421515; doi:10.2196/12369)
Supplement: Multimedia Appendix 4 [file jmir_v21i3e12369_app3.pdf]

Generalized additive model (GAM) plots showing the relationship between mean blood pressure (MBP) and (A) the hourly averaged temperature; (B) the temperature difference between the maximum and minimum in the 12 hours prior to blood pressure measurement; (C) the hourly averaged relative humidity; and (D) the hourly averaged wind speed. The effect of temperature (both hourly averaged temperature and the temperature difference within the 12 hours) on MBP was linear, while the effect of relative humidity and wind speed on MBP was nonlinear. The effect of the temperature difference on MBP was statistically significant in the multivariate regression model (contrast to the model for systolic blood pressure, in which the effect of temperature difference was not statistically significant).

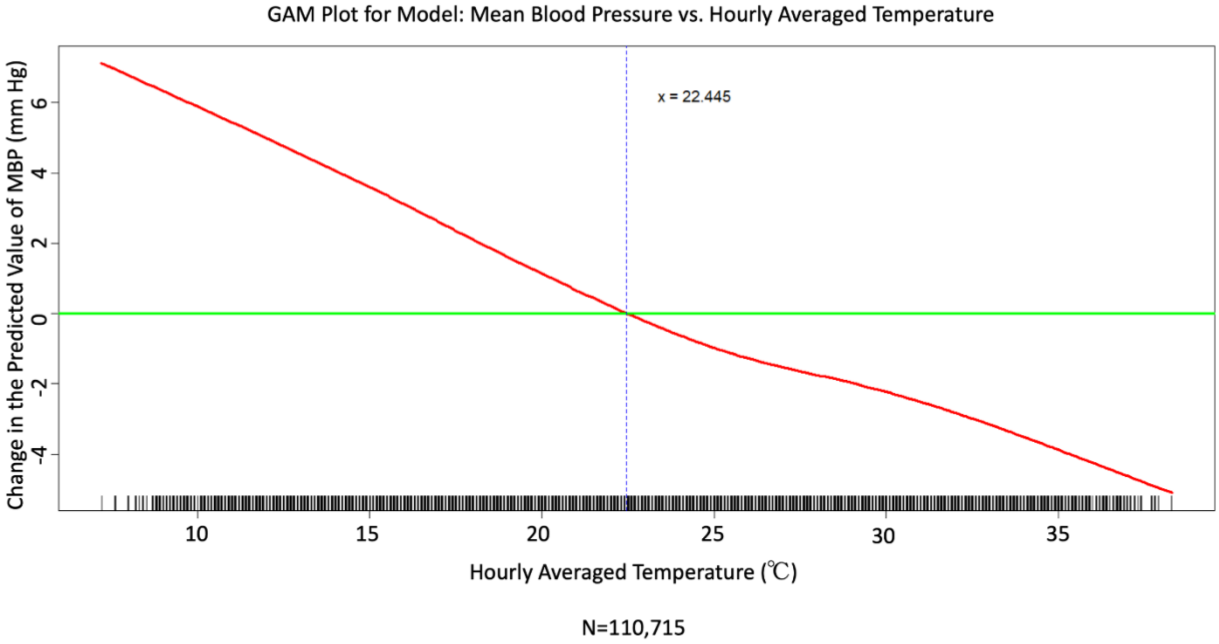

A. The relationship between MBP and hourly averaged temperature

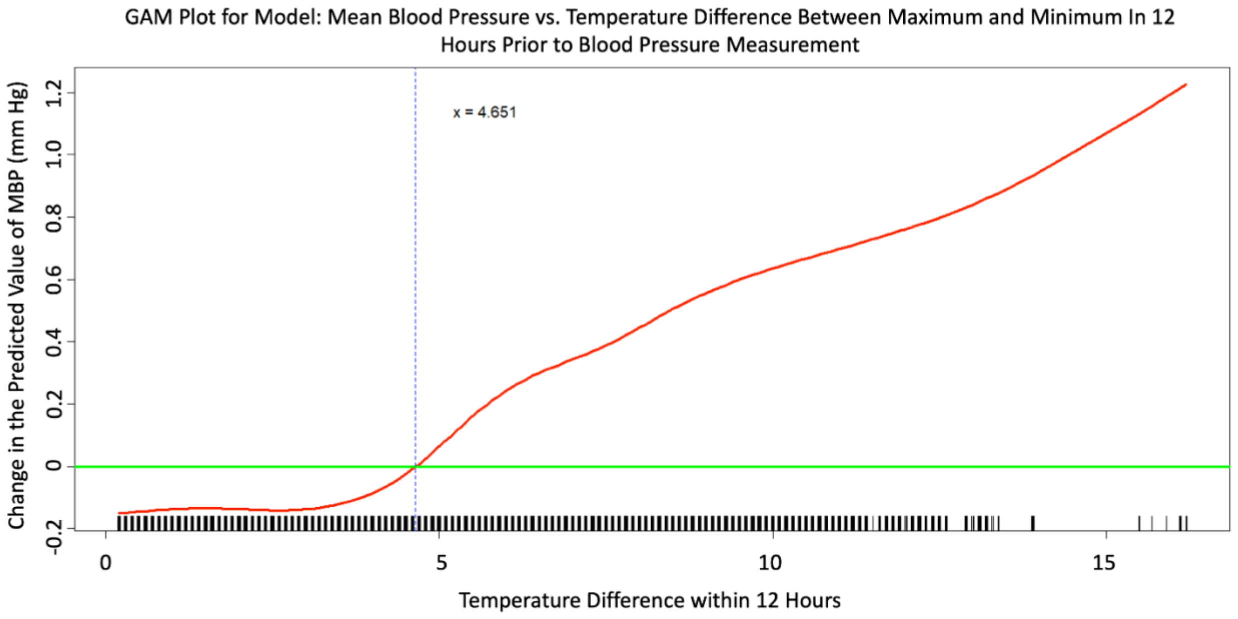

N=110,715

B. The relationship between MBP and temperature difference between maximum and minimum in 12 hours prior to blood pressure measurement

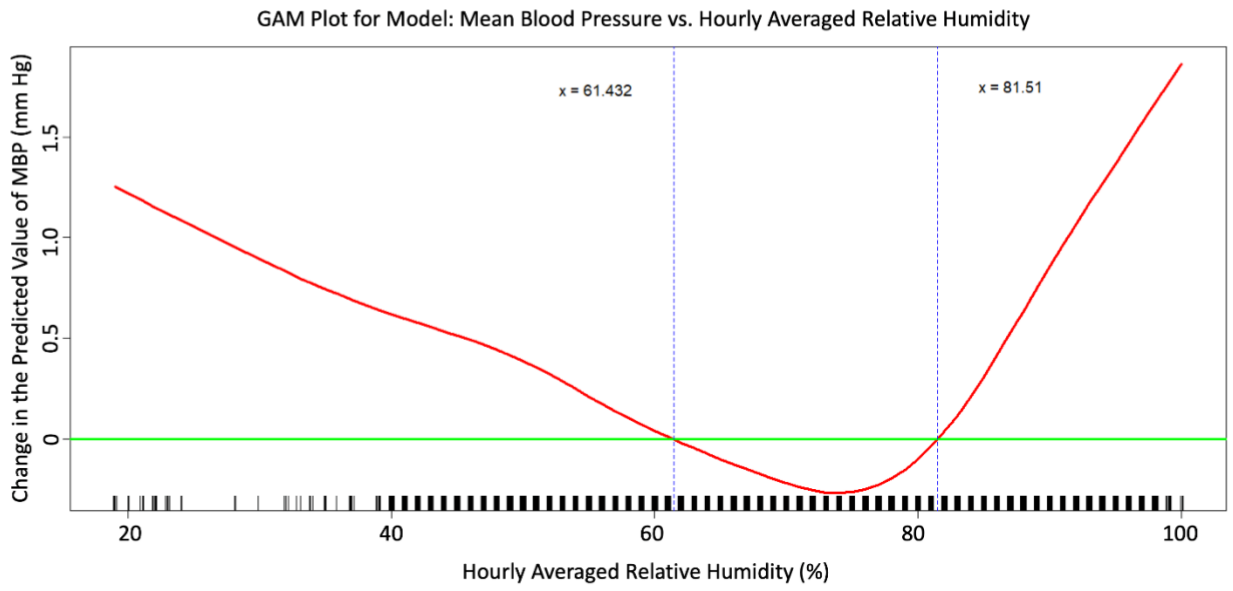

N=110,715

C. The relationship between MBP and hourly averaged relative humidity

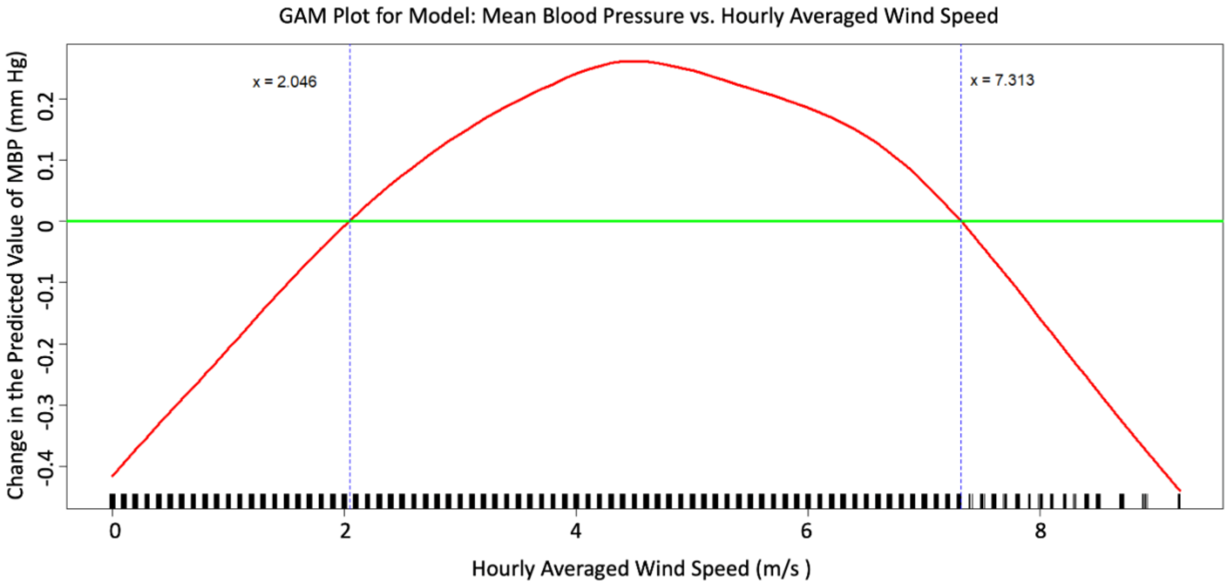

N=110,715

D. The relationship between MBP and hourly averaged wind speed
